# Sex-related differences in visuomotor skill recovery following concussion in working-aged adults

**DOI:** 10.1186/s13102-022-00466-6

**Published:** 2022-04-20

**Authors:** Nicole Smeha, Ravneet Kalkat, Lauren E. Sergio, Loriann M. Hynes

**Affiliations:** 1 School of Kinesiology and Health Science, York University, 357 Bethune College, 4700 Keele Street, Toronto, ON M3J 1P3 Canada; 2 York University Sport Medicine Team, York University, Toronto, Canada; 3 Centre for Vision Research, York University, Toronto, Canada

**Keywords:** Eye-hand coordination, Sex differences, Concussion, Human, Motor control, Psychophysics, Recovery

## Abstract

**Background:**

The ability to perform visually-guided motor tasks requires the transformation of visual information into programmed motor outputs. When the guiding visual information does not align spatially with the motor output, the brain processes rules to integrate somatosensory information into an appropriate motor response. Performance on such rule-based, “cognitive-motor integration” tasks is affected in concussion. Here, we investigate the relationship between visuomotor skill performance, concussion history, and sex during the course of a post-concussion management program.

**Methods:**

Fifteen acutely concussed working-aged adults, 11 adults with a history of concussion, and 17 healthy controls all completed a recovery program over the course of 4 weeks. Prior to, mid-way, and following the program, all participants were tested on their visuomotor skills.

**Results:**

We observed an overall change in visuomotor behaviour in all groups, as participants completed the tasks faster and more accurately. Specifically, we observed significant visuomotor skill improvement between the first and final sessions in participants with a concussion history compared to no-concussion-history controls. Notably, we observed a stronger recovery of these skills in females.

**Conclusions:**

Our findings indicate that (1) concussion impairs visuomotor skill performance, (2) the performance of complex, rule-based tasks showed improvement over the course of a recovery program, and (3) stronger recovery in females suggests sex-related differences in the brain networks controlling skilled performance, and the effect of injury on these networks.

**Supplementary Information:**

The online version contains supplementary material available at 10.1186/s13102-022-00466-6.

## Background

Concussion is a form of mild traumatic brain injury (mTBI) induced by biomechanical forces that results in a complex pathophysiological condition affecting the brain [1, 2]. An impulsive blow to the head or body triggers this transient neurologic syndrome and produces a constellation of physical and cognitive symptoms, and at times, a loss of consciousness [2, 3]. Concussion presents a significant health problem and public health concern: 1.6–3.8 million sport-related concussions alone are reported annually in the United States, while it is estimated that 110 per 100,000 Canadians sustain a concussion annually [1, 4, 5]. Many patients suffering from concussion experience a gradual resolution of signs and symptoms over three months, although complete recovery can be experienced in the majority of cases by two weeks post-injury [2, 6]. Despite the optimistic prognosis, some individuals do not recover within this expected timeframe and are described as experiencing persistent symptoms [7]. These symptoms may interfere with one’s quality of life and lead to disability.

Current recommendations prescribe patients complete physical and cognitive rest for an undefined period of time until symptoms subside, followed by a sequence of physical and cognitive tests before being cleared to return to their daily activities [1]. The separate evaluation of cognitive and motor domains is of significant concern: several tasks we engage in on a daily basis, including the successful use of a computer, driving a car, and participating in sports, require the integration of cognition and motor action, an ability known as cognitive-motor integration (CMI) [8]. In these tasks, the guiding visual information is spatially decoupled from the arm movement, and as a result, the integration of context specific rules into the planning of reaching movements is necessary [9, 10]. Thus, successful integration of thought and action is essential to successful interaction with our environments [11–14]. This interaction is contingent on our brain’s ability to integrate sensory and motor information in order to execute an efficient motor plan—in other words, we often need to be able to think and move at the same time. The performance of tasks requiring CMI is dependent on intact connections between frontal, parietal, and subcortical brain regions [9, 15, 16]. Previous research has shown that following concussion, the integrity of these networks may be compromised, resulting in an impaired ability to integrate rules into coordinated motor tasks [17–21]. However, patients may be cleared to return to their activities before CMI abilities have recovered [17, 18]. Sex-related differences have also been observed in the brain networks which control CMI [22], and sex-related differences have been observed more generally in tasks requiring eye-hand coordination [16, 23]. An increasing body of research is providing evidence for sex-related differences in concussion rates, symptoms, and recovery trajectory [24–31]. Lastly, the vast majority of this work has been in the realm of sport-related concussion in youth and young adults [32–34]. Examining the utility of a concussion management program on CMI skill recovery, and sex-related differences in this recovery, would be useful in order to fill the knowledge gap around concussion management for a broader demographic sample.

Ensuring that skilled performance has recovered following a concussive injury is important in that it will decrease vulnerability to another injury, and return an individual to their previous quality of daily life. The main aim of the present study was, therefore, to examine the recovery of CMI performance in 3 groups (acutely concussed working-aged adults, asymptomatic adults with a prior history of concussion, and healthy no-concussion-history controls) during the course of a post-concussion management program. A secondary aim of this study was to examine whether sex- and age-related differences were related to CMI performance over the duration of the program. As an exploratory aspect to the study, the relationship between CMI performance and symptom recovery was examined.

## Methods

### Participants

Fifteen adults with an acute concussion (mean age 27.33 $\pm$ 6.94; 10 females), 11 adults with a history of concussion (mean age 28.27 $\pm$ 7.30; 7 females), and 17 healthy controls (mean age 23.24 $\pm$ 4.01; 8 females, 1 unidentified) participated in this study. We obtained the information about concussion history and demographic data from all participants by our own and established questionnaires (SCAT5). Characteristics of participants detailing concussion history and demographic data are summarized in Table 1. All participants were recruited from local community clinics and through posters placed around York University’s campus. Participants were included in the acute concussion group if they were symptomatic or were between 10- and 125-days post-injury, and all acute concussions were physician diagnosed. This reference range was selected because of the timeframe during which symptom resolution is expected to occur in most individuals suffering from concussion, while also attempting to capture individuals with lingering symptoms. Individuals were included in the concussion history group if they reported a history of concussion of greater than 125 days prior to beginning the active recovery program and were asymptomatic. All participants had normal or corrected-to-normal vision. Participants were included in the healthy group if they reported no history of concussion. All individuals with cerebrovascular or cardiovascular disease, chronic headache, diabetes, cancer and clinical depression were excluded. Delivery of concussion management therapies and CMI testing took place at York University in Toronto, Ontario, Canada, between 2018 and 2019. The study protocol was approved by York University’s Ethics Review Board and conformed to the standards of the Canadian Tri-Council Research Ethics guidelines. Participants signed written informed consent forms before participating in this study.

**Table 1:** Participant demographics

	Healthy, no history of concussion	Asymptomatic, with previous concussion history	Symptomatic, acutely concussed
N	17	11	15
Sex			
Female	8	7	10
Male	8	4	5
Unreported	1	–	–
Mean age in years (SD)	23 (4.01)	28 (7.30)	27 (6.94)
Median age in years (range)	23 (19–36)	28 (18–40)	29 (18–36)
Mean days since last concussion (SD)	–	1837.1 (2675.79)	46 (34.25)
Contact/limited contact sports	16	10	12
Non-contact sport	9	5	3
Mean minimum time playing sports in years (SD)	12.27 (5.57)	14.33 (9.76)	14.22 (8.51)
Employment status			
Student	7	2	1
Part-time	6	4	3
Full-time	3	5	5

Characteristics for 15 acutely concussed participants, 11 participants with a concussion history, and 17 participants with no history of concussion

### Concussion management program

**Fig. 1:**
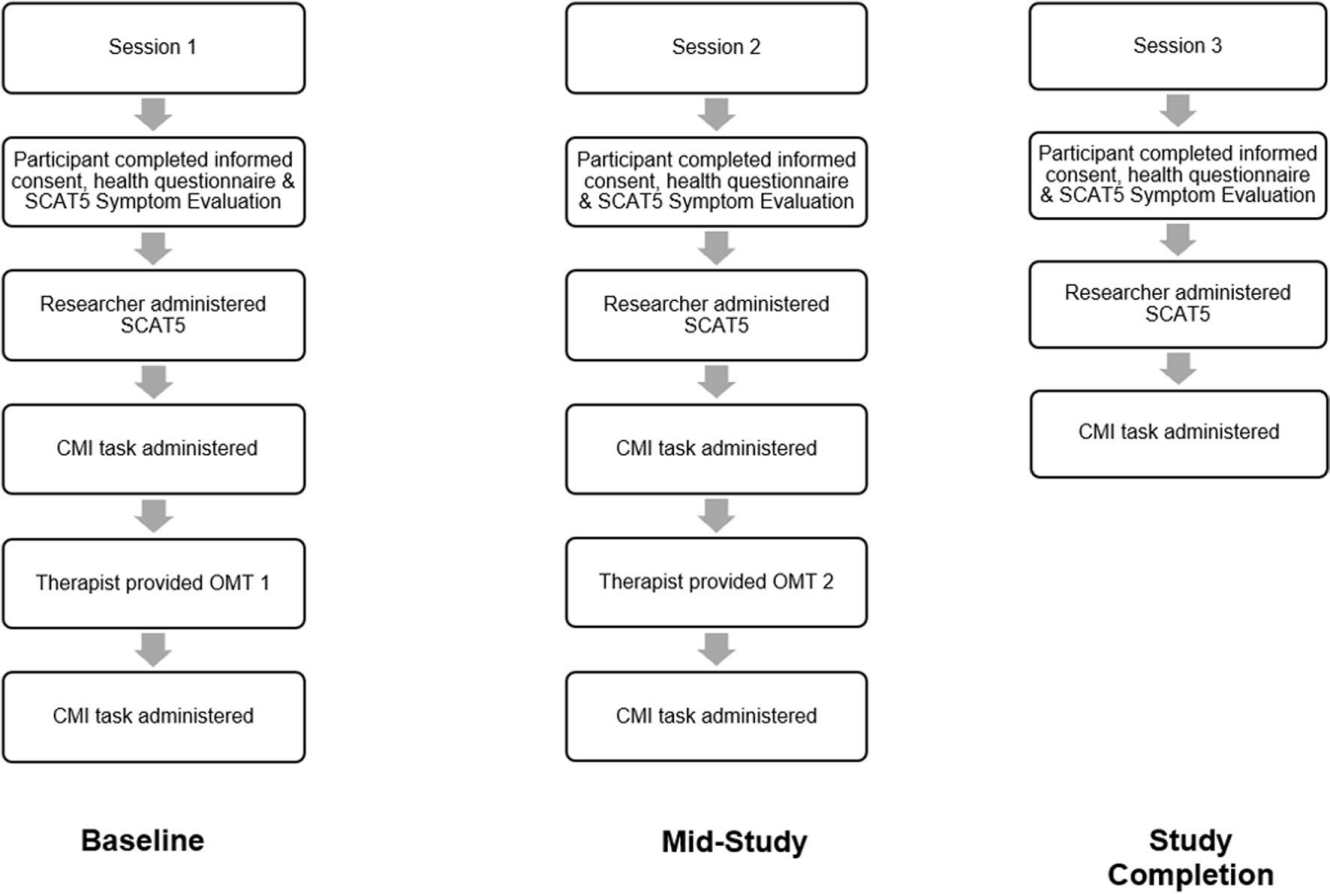
Research procedure flow diagram

Participants attended 3 sessions: 2 treatment sessions and a follow-up data session over the course of 6 weeks (1 session/2 weeks) (Fig. 1). To test for concussion symptomatology, participants completed a health questionnaire in addition to a self-administered form (the SCAT5 Symptom Evaluation) and were administered the SCAT5 by a researcher. To test cognitive-motor abilities, participants were tested on two visuomotor transformation tasks. Participants were then provided osteopathic manual treatment (OMT), a form of manual therapy which has been shown to be effective in concussion management [35–37]. Within a treatment session, participants were retested on the visuomotor tasks after manual therapy. During the final session, the same data collection procedures were followed, without the manual therapy.

### Procedures

**Fig. 2:**
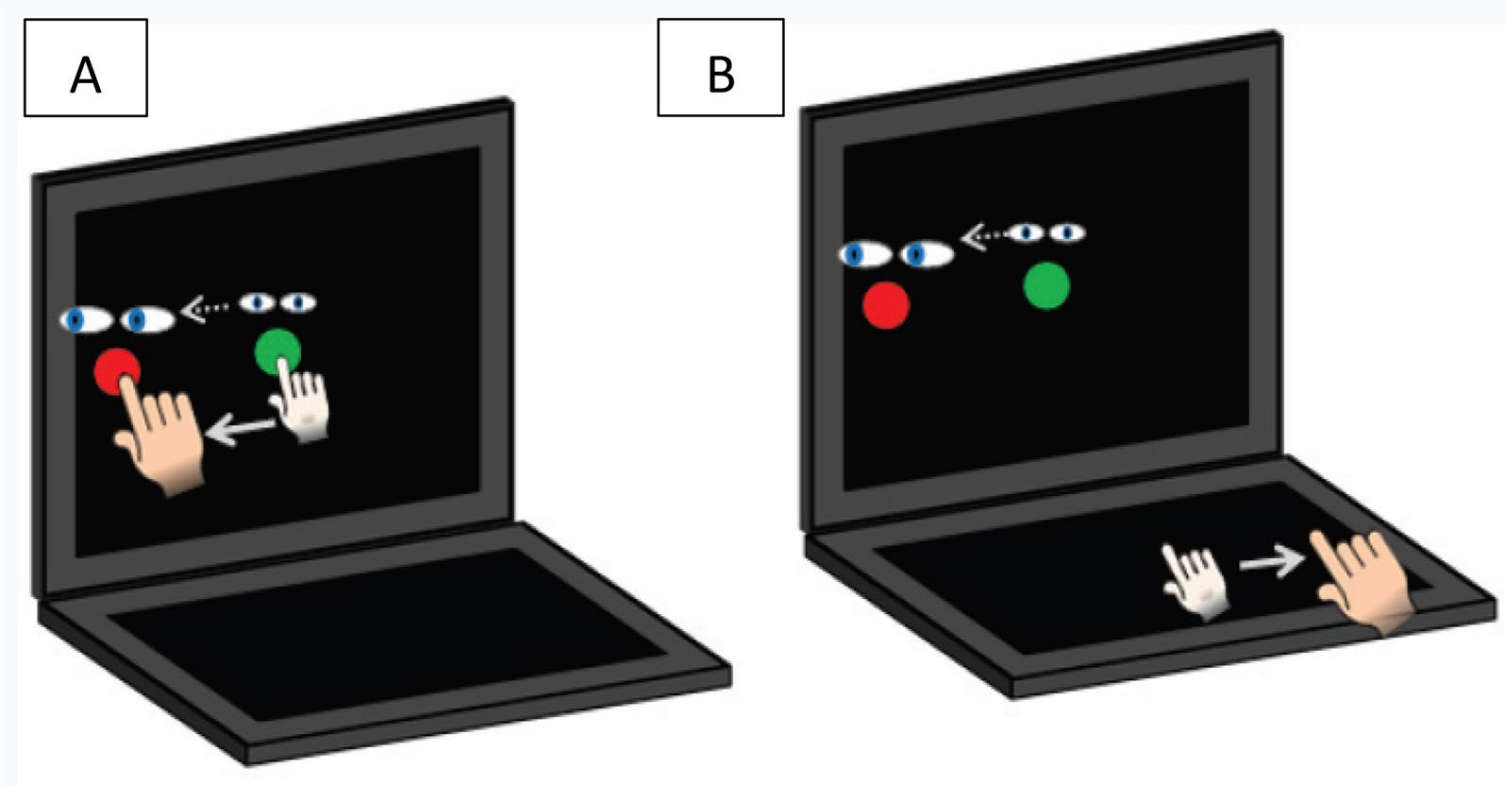
Illustration of the cognitive-motor integration task. The green circle denotes the central home target in which all movements begin. A red target appears in one of 4 peripheral direction (top, bottom, left, or right of centre) after 2000 ms which serves as the ‘Go’ cue. **A** The standard (ST) condition, in which eye and arm movements are congruent (moved to the same peripheral target). **B** The non-standard cognitive-motor integration (CMI) condition, in which vision and movement are decoupled to a plane dissociation (eyes look at the vertical screen while hand moves along the horizontal screen), and visual feedback reversal (cursor movement 180° rotated from hand motion)

Participants performed two visuomotor transformation tasks that required sliding the index finger of the dominant hand directly along an ASUS touchscreen tablet (Asus Transformer Book T100TAF) or on an externally connected USB touchpad (Keytec™, TycoTouch, USA) situated perpendicular (in the horizontal plane) to the ASUS screen. These methods have been described in our previous work using this task [10–13]. To summarize, the standard condition was the standard visuomotor mapping task, where the spatial location of the viewed target and the required movement were in alignment (hand movements were made on the ASUS tablet directly to the peripheral target) (Fig. 2). The CMI condition was a non-standard visuomotor task that included two levels of decoupling. Participants were instructed to maintain their eye focus on the vertically oriented tablet while manipulating a cursor on the horizontally oriented Keytec™ touchpad. The feedback for this task was also rotated 180°: for instance, to move the cursor to the left, the participant was required to slide their finger to the right.

The central green target appeared in the center of the screen for each trial and had a diameter of 7.5 mm. Prior to initiation of the trial, participants were instructed to place their finger in the center of the green target. After a delay period of 2000 ms, a red peripheral target was presented 55 mm away from the center (up, down, left, right). This served as the ‘Go’ signal for the participant to slide their finger along the screen directly to the presented peripheral target. After reaching the peripheral target and remaining there for 500 ms, the peripheral target disappeared. This served as the signal for the end of the trial. Following a delay of 2000 ms, the central target reappeared, signaling the participant to return to the center for the next trial. The same sequence was followed for the CMI condition, but participants were sliding their fingers on the horizontal touchpad in the opposite direction of the presented target. Each participant completed 20 trials per condition (5 trials to each peripheral target), prior to and following manual therapy. Trials to the peripheral targets and condition order were presented in a randomized order. In both conditions, participants were instructed to move as quickly and as accurately as possible.

### Data processing and analysis

Kinematic measures, including timing, finger position and error data were recorded for each trial and converted into a binary readable format using a custom written C++ application. Unsuccessful trials were detected by the data collection software and resulted in trial termination if the finger left the home target too early (< 2000 ms), reaction time (RT) was too short (< 150 ms), RT was too long (> 8000 ms), or movement time was too long (> 10,000 ms). Trials in which the first ballistic movement exited the boundaries of the center target in the wrong direction (greater than 45° from a straight line to target) were coded as direction reversal errors, and analyzed as separate variables from the correct trials. A custom-written analysis program (using Matlab, Mathworks, Inc., USA) was used to generate a computerized velocity profile of each trial’s movement, with movement onset and end being recorded at 10% peak velocity. These profiles were then verified by visual inspection, and corrections were performed when necessary. The scored data was processed to compute 7 different movement timing and execution outcome measures [10–13].

### Dependent measures

The dependent variables of interest were reaction time, movement time, path length, peak velocity, percent of trials resulting in a direction reversal, and movement accuracy and precision. The different variables are described in detail in the following section.

#### Movement timing variables

Reaction time (RT) was calculated as the time interval (milliseconds; ms) between the central target disappearance and movement onset. Movement time (MT) was calculated as the time in ms between movement onset and offset, and is composed of total movement (MTf, full movement offset) as well as ballistic movement (MTb, initial movement offset). If no corrective movements were made, ballistic movements were equivalent to full movement trajectories. To compare movement timing differences in the standard and CMI conditions, performance in the standard condition was subtracted from performance in the CMI condition ($\Delta$RT; $\Delta$MT, calculated using MTf) at each time point. Peak velocity (PV) was the maximum velocity in mm/ms obtained for each trial.

#### Pathlength

The ballistic pathlength (PLb) was recorded as the distance in mm between the starting position and the endpoint of the first ballistic movement (i.e., distance covered during the ballistic movement time). Full pathlength (PLf) was measured as the distance between start to final end location of the entire movement. Movements comprised of curves or deviations from a straight path between the central and peripheral target would thus result in a longer pathlength*.*

#### Endpoint analysis

Absolute error (AE), the end-point accuracy, was the average distance from the individual movement endpoints to the actual target location, in mm. Variable error (VE), the end-point precision, describes the distance between the individual movement endpoints (σ2) from their mean movement, measured in mm.

#### Direction reversals

Direction reversals were only applicable in the CMI condition. They were calculated as the percentage of total trials that constituted a deviation of greater than ± 45° from a straight line between the center of the central and peripheral targets (%DRs).

### Statistical analysis

Our statistical approach was to perform univariate and bivariate analyses in order to assess differences between groups and to look for possible significant differences in the dependent variables as a function of concussion history and sex, followed by multivariate analyses to examine the relationship between exposure variables of interest (age, concussion history, and sex) and the dependent variables, while controlling for the effect of these variables in the model.

Trials containing outcome measures > 2 standard deviations (SDs) away from the mean for a given condition in a given participant were considered outliers and removed before statistical analysis. All remaining data were checked for normal distribution and sphericity (Mauchly’s test), and were Greenhouse–Geisser corrected where necessary. Statistical analyses were performed using SPSS statistical software (IBM Inc.). Statistical significance levels were set to α $\le$ 0.05.

Descriptive statistics were used to summarize subjects’ characteristics. A frequency distribution was conducted on group (acute concussion, concussion history, healthy), sex, and recovery program completion. The mean and standard deviation of age was also determined. The normal distribution of all dependent variables was assessed, and the mean and standard deviation of each measure of visuomotor performance was determined.

#### Visuomotor task performance variables

For all dependent measures (RT, MTf, MTb, $\Delta$RT, $\Delta$MT, PV, PLf, PLb, AE, VE, %DRs) in both conditions, effects of group (acute concussion, concussion history, healthy) and sex were analyzed separately using a repeated-measures mixed ANOVA, to test for any concussion- or sex-related behavioural differences. When there were significant main or interaction effects, pair-wise comparisons were used. Findings in either sex or concussion history were used to develop the models for linear regression. Note that for this study we constrained our analyses to a comparison of the behavioural data collected at the beginning of each treatment session. That is, the repeated measures analysis compared data across treatment sessions, rather than pre-post analysis within sessions.

#### Relationship between visuomotor task performance and participant characteristics

Blocked hierarchical linear regression analysis was used to determine if any of the independent variables can predict improvements in visuomotor performance. To investigate whether possible visuomotor performance deficits were related to concussion history, sex, or age over the course of an active recovery program, we correlated the dependent main variables of the standard condition and the CMI condition with these factors using a linear regression analysis. We were specifically interested in whether total number of concussions sustained (0, 1, 2, 3 or more; either acute or past history) was related to visuomotor performance. Therefore, the acute concussion and concussion history groups were combined for the purpose of this analysis. Participants who did not complete the recovery program were excluded from this analysis. A ‘pre-post’ score was used in the linear regression, in which the final dependent measure score was subtracted from the initial score in order to measure changes in visuomotor performance prior to and following the program. A larger difference is indicative of improved performance.

#### Relationship between CMI performance and concussion symptom recovery

The relationship between CMI performance and number of concussion symptoms and severity (assessed using the SCAT5) was analyzed using Pearson’s correlation. As this was an exploratory analysis, only healthy and acutely concussed participants were included.

## Results

Table 1 presents selected characteristics of the participants of this study. Of the 43 participants, 17 were healthy (40%), 11 were asymptomatic with a history of concussion (26%), and 15 were acutely concussed (35%). The total sample had a somewhat higher percentage of females (58%), and the mean age was 25.95. The median age of participants was 23, with a range of 18–40 years old. All participants but one reported participating in contact or non-contact sports at either a recreational or competitive level, and 14 participants reported engaging in both. Sports that require contact as a regular part of strategy or play were categorized as contact or limited contact sports (such as basketball, hockey, or rugby), while sports in which players do not have physical contact with each other (such as tennis or swimming) were classified as non-contact sports [38–40]. Ten participants were students, 13 reported part-time employment, and 13 reported full-time employment.

### Group comparisons

**Fig. 3:**
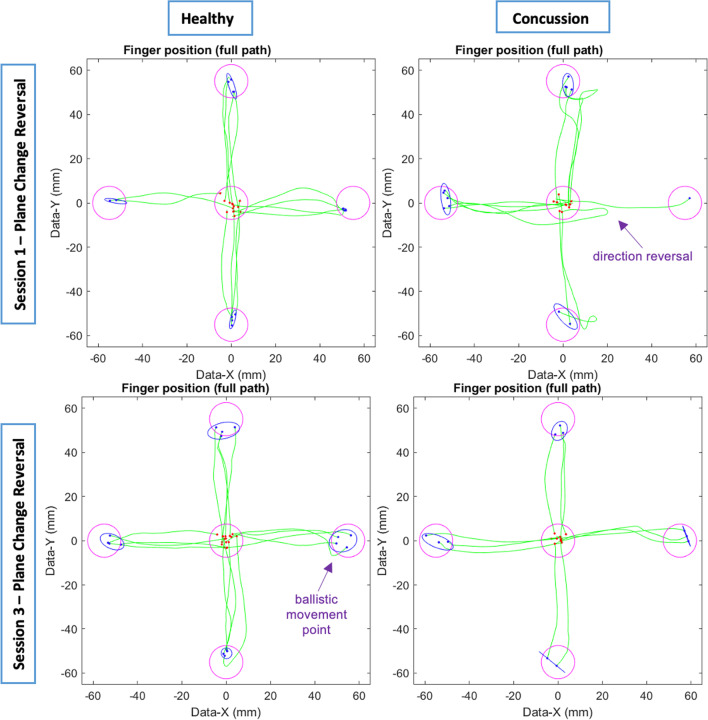
Examples of typical full hand movement trajectories for both healthy (left) and concussed (right) individuals, at start (top) and end (bottom) of the program. Trajectories begin at the central target (red dots) and move towards one of four peripheral targets, where each green line represents a single movement trajectory. Blue ellipses denote the 95% C.I. for the final end point of the finger movements (blue dots). Correct trials (green lines) are shown. Any target with less than 5 trajectories indicates error trials (not shown). Ballistic measures are taken from initial slowing point (“ballistic movement point”, < 10% peak velocity)

Visuomotor skilled performance differences were observed between the concussion group and the healthy and history groups over the course of the recovery program. Examples of full hand movement trajectories in healthy and acutely concussed participants in the CMI condition are plotted in Fig. 3. The descriptive statistics for visuomotor performance in the standard condition during each session are summarized in Table 2, in addition to statistical outcomes of the repeated-measures mixed ANOVA for group (healthy, previous concussion history, acutely concussed). The descriptive statistics for the non-standard condition during each session along with statistical outcomes of the repeated-measures mixed ANOVA for group are summarized in Table 3.

**Table 2:** Descriptive statistics of visuomotor performance by group and statistical outcomes of repeated-measures mixed ANOVA in the standard condition

Parameter	Session number	Healthy, no history of concussion	Asymptomatic, with previous concussion history	Symptomatic, acutely concussed	Repeated-measures mixed ANOVA outcomes		
					Session number	Group	Session number × Group
AE (mm)	1	2.64 ± 0.67	2.44 ± 0.53	3.60 ± 0.70	F = 2.73	F = 2.27	F = 2.57*
	2	3.36 ± 0.92	3.11 ± 0.78	3.20 ± 0.91			
	3	3.36 ± 0.92	3.00 ± 0.50	3.40 ± 0.84			
VE (mm)	1	2.50 ± 0.53	2.00 ± 0.71	2.70 ± 0.48	F = 2.18	F = 1.18	F = 1.61
	2	2.40 ± 0.70	2.56 ± 0.88	2.80 ± 0.63			
	3	1.90 ± 0.88	2.44 ± 0.88	2.30 ± 0.95			
RT (ms)	1	357.30 ± 42.23	352.43 ± 32.65	364.29 ± 114.49	F = 1.19	F = 0.01	F = 0.38
	2	343.40 ± 52.12	345.71 ± 37.56	345.00 ± 45.04			
	3	354.00 ± 39.46	347.43 ± 49.26	336.86 ± 47.23			
MTb (ms)	1	404.64 ± 75.28	420.29 ± 90.74	407.89 ± 92.27	F = 2.71	F = 0.09	F = 1.20
	2	424.36 ± 95.20	430.00 ± 109.40	374.44 ± 77.64			
	3	435.00 ± 82.74	457.57 ± 135.11	541.67 ± 354.87			
MTf (ms)	1	494.00 ± 95.85	532.57 ± 114.20	479.67 ± 102.57	F = 1.34	F = 0.68	F = 1.41
	2	477.70 ± 78.79	548.43 ± 126.06	472.44 ± 103.44			
	3	482.90 ± 116.98	544.14 ± 110.00	654.78 ± 396.82			
PLb (mm)	1	52.50 ± 1.18	52.78 ± 1.72	52.60 ± 1.27	F = 0.39	F = 6.13**	F = 2.99*
	2	51.80 ± 1.48	53.89 ± 1.69	52.60 ± 1.26			
	3	51.90 ± 1.73	55.00 ± 2.83	52.00 ± 1.76			
PLf (mm)	1	54.91 ± 1.64	54.56 ± 1.59	53.50 ± 1.51	F = 0.50	F = 3.20	F = 1.84
	2	53.82 ± 1.66	56.22 ± 3.19	54.10 ± 1.66			
	3	53.45 ± 1.44	57.56 ± 7.43	54.00 ± 2.94			
PV (mm/ms)	1	145.09 ± 24.56	128.83 ± 8.38	149.00 ± 30.91	F = 0.34	F = 1.20	F = 0.03
	2	143.82 ± 21.34	132.50 ± 19.93	147.89 ± 48.25			
	3	141.00 ± 28.46	125.67 ± 17.19	143.22 ± 33.90			

*p < 0.05; **p < 0.01; ***p < 0.001

**Table 3:** Descriptive statistics of visuomotor performance in the non-standard condition by group and statistical outcomes of repeated-measures mixed ANOVA for the non-standard condition

Parameter	Session Number	Healthy, no history of concussion	Asymptomatic, with previous concussion history	Symptomatic, acutely concussed	Repeated-Measures Mixed ANOVA outcomes		
					Session number	Group	Session number × Group
%DRs	1	4.46 ± 5.97	8.60 ± 8.42	21.90 ± 17.62	F = 3.81*	F = 5.20	F = 2.65*
	2	4.46 ± 6.20	5.66 ± 5.24	14.65 ± 20.27			
	3	6.53 ± 6.42	3.70 ± 5.25	8.80 ± 5.67			
AE (mm)	1	3.54 ± 0.52	4.00 ± 0.94	4.30 ± 0.68	F = 4.91*	F = 1.27	F = 1.31
	2	4.27 ± 0.69	4.05 ± 0.46	4.46 ± 0.79			
	3	4.38 ± 0.77	4.40 ± 0.97	4.50 ± 0.85			
VE (mm)	1	3.00 ± 0.60	3.00 ± 0.47	2.89 ± 0.78	F = 3.32*	F = 1.00	F = 0.72
	2	3.30 ± 0.63	3.27 ± 0.52	3.54 ± 0.44			
	3	2.75 ± 1.29	2.80 ± 0.42	3.33 ± 0.71			
RT (ms)	1	496.17 ± 128.57	528.80 ± 73.33	557.22 ± 94.35	F = 16.81***	F = 0.54	F = 1.38
	2	466.50 ± 87.10	488.20 ± 76.34	504.44 ± 56.99			
	3	454.25 ± 100.93	483.00 ± 83.23	459.79 ± 67.70			
MTb (ms)	1	686.77 ± 143.31	862.60 ± 309.03	832.50 ± 218.77	F = 12.04*	F = 1.11	F = 2.09
	2	637.62 ± 168.08	713.10 ± 251.76	796.60 ± 278.47			
	3	638.62 ± 149.32	659.70 ± 210.39	668.10 ± 192.54			
MTf (ms)	1	930.15 ± 149.07	1100.20 ± 376.38	1136.78 ± 352.19	F = 12.25***	F = 0.46	F = 1.38
	2	854.15 ± 243.00	940.60 ± 257.48	925.22 ± 313.39			
	3	862.69 ± 322.05	852.90 ± 281.46	871.56 ± 313.06			
PLb (mm)	1	50.15 ± 3.58	51.20 ± 2.57	53.70 ± 6.00	F = 0.80	F = 1.13	F = 0.90
	2	52.27 ± 2.72	52.25 ± 2.68	53.07 ± 4.53			
	3	52.00 ± 2.97	52.20 ± 1.99	52.80 ± 4.18			
PLf (mm)	1	57.00 ± 6.21	57.00 ± 2.94	61.56 ± 8.25	F = 1.12	F = 0.31	F = 1.38
	2	57.22 ± 5.68	57.67 ± 4.26	55.92 ± 4.01			
	3	57.46 ± 8.34	55.70 ± 3.37	57.33 ± 5.83			
PV (mm/ms)	1	93.62 ± 20.39	81.50 ± 26.57	90.20 ± 24.82	F = 9.82**	F = 0.23	F = 1.52
	2	103.70 ± 26.90	102.30 ± 37.24	89.35 ± 33.30			
	3	106.38 ± 25.93	109.00 ± 40.85	101.60 ± 26.81			
$\Delta$MT (ms)	1	405.45 ± 186.99	291.89 ± 512.85	902.60 ± 826.00	F = 4.63*	F = 1.98	F = 3.28*
	2	371.27 ± 159.87	253.89 ± 283.47	542.10 ± 394.71			
	3	363.73 ± 240.35	255.78 ± 174.69	236.40 ± 501.14			

*p < 0.05; **p < 0.01; ***p < 0.001

#### Movement timing variables

**Fig. 4:**
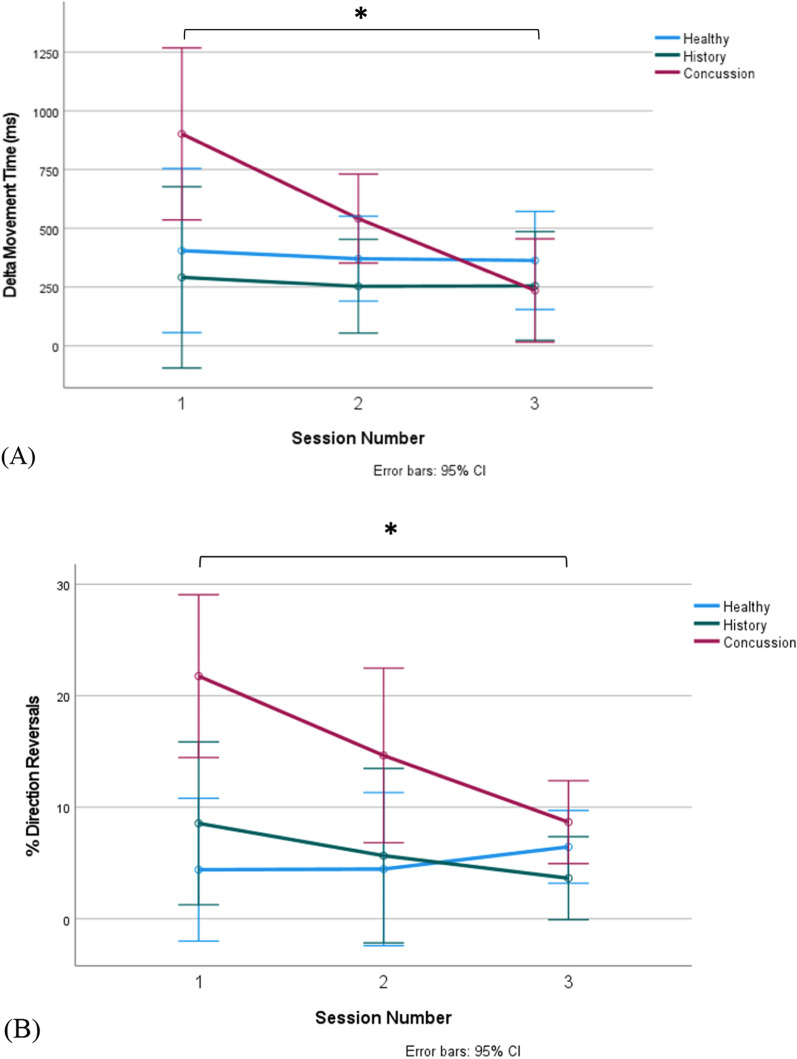
**A** Percent of trials in the CMI condition resulting in direction reversals, by session and group. The percent of direction reversals is significantly greater between the concussion group and the healthy and history groups during session 1. Following the program time, the concussion group experiences a significant improvement. **B** Difference in Movement Time in the CMI and Standard Condition, by session and group. The difference is significantly greater between the concussion group and the healthy and history groups during session 1 (delta MT). Following the program time, the concussion group experiences a significant improvement. *p < 0.05

A repeated-measures mixed ANOVA revealed a statistically significant main effect of session number on MTb (F = 12.04, df = 2, p < 0.001, η^2^ = 0.29), MTf (F = 12.25, df = 2, p < 0.001, η^2^ = 0.30), RT (F = 16.81, df = 2, p < 0.001, η^2^ = 0.38) and PV (F = 9.82, df = 1.43, p = 0.001, η^2^ = 0.25) in the CMI condition. Specifically, reaction time and ballistic and full movement times decreased across the sessions, and peak velocity increased across the sessions in all groups. To compare performance differences between the standard and CMI conditions, a difference score was calculated for the performance timing variables (CMI—Standard condition, calculated for all 3 sessions). The repeated-measures ANOVA on $\Delta$MT revealed a significant effect of session number (F = 4.63, df = 2, p = 0.03), with a main effect of session number × group (F(2.49, 33.59) = 3.28, p = 0.04). The session number × group interaction revealed that decreases in $\Delta$MT were driven by the concussion group: the healthy and history groups maintained a similar $\Delta$MT throughout the recovery program, while the concussion group experienced a decrease in $\Delta$ MT by the final session (Fig. 4a).

#### Absolute error

Repeated-measures mixed ANOVA revealed a significant session number × group interaction for AE (endpoint accuracy) in the standard condition (F(4, 54) = 2.57 = 0.048, η^2^ = 0.16), and a significant effect of session number in the CMI condition (F = 4.91, df = 2, p = 0.01, η^2^ = 0.14). In the standard condition, there was an increase in AE across the sessions, and a significant difference was identified between the history and concussion groups. Fisher’s least significant difference (LSD) post-hoc analysis revealed that the concussion group had consistently larger AE scores across the 3 sessions compared to the history group (0.55 mm, p = 0.04). In the CMI condition, there was a significant increase in AE across the sessions. However, since participants remained within the target, the increases in AE reflect participants ending their movements once directly inside the target, rather than aiming for the target center (from which accuracy was measured), rather than a worsening performance.

#### Direction reversals

A significant main effect of session number and a significant session number X group interaction was found for %DRs in the CMI condition. Overall, a significant decrease in the %DRs was identified across the sessions (F = 3.81, df = 2, p = 0.028; η^2^ = 0.11), though the interaction effect revealed that the observed decrease was driven by the concussion group (F(4, 60) = 2.65, p = 0.042, η^2^ = 0.15). The healthy and history groups maintained similar %DRs in the CMI condition across the sessions, while the concussion group experienced a significant decrease in the percentage of reversal trials where they started off in the wrong direction, by the final session (p = 0.042). Despite the decrease, the concussion group still performed more %DRs by the end of the program (Fig. 4b). The LSD post-hoc test revealed that the concussion group had an average 9.96% more DRs compared to the healthy group across the 3 sessions (p = 0.005), and an average 9.13% more DRs compared to the history group across the 3 sessions (p = 0.015).

### Sex-related comparisons

#### Movement timing variables

Repeated-measures mixed ANOVA using session number as a main effect revealed significant differences in the CMI condition across the sessions for MTb (F = 10.81, df = 2, p < 0.001; η^2^ = 0.27), MTf (F = 11.67, df = 1.65, p < 0.001; η^2^ = 0.29), RT (F = 14.33, df = 2, p < 0.001, η^2^ = 0.34) and PV (F = 9.06, df = 1.41, p = 0.002; η^2^ = 0.23). In both sexes, a similar decrease in these variables was observed across all 3 sessions for MTb, MTf, and RT, while an increase was observed for PV. A significant session number X sex interaction was also observed for full movement time **(**F(1.65, 47.81) = 4.20, p = 0.03, η^2^ = 0.13). Females experienced a more significant decrease in MTf compared to males (p < 0.001).

#### Absolute error

An ANOVA using session number as a main effect also revealed a significant effect of session number for AE in the standard condition (F = 3.62, df = 2, p = 0.03; η^2^ = 0.12) and in the CMI condition (F = 4.52, df = 2, p = 0.03; η^2^ = 0.14). A significant increase in AE across the sessions occurred in both conditions. Again, this likely indicates participants learning to stop immediately upon entering the target zone, rather than a worsening of performance. No interaction effects were observed between session number X sex.

Results of the ANOVAs revealed that session number (e.g., time) was the greatest factor affecting visuomotor performance, although group differences in concussion history emerged. Notably, more changes were observed in the concussed group on CMI tasks over time compared to the healthy and history groups. This supports the notion that these changes were related to neural recovery from concussion rather than learning effects, and that concussion may affect performance on visuomotor tasks. For this reason, concussion history was chosen as a factor to include in the linear regression analysis. Though no significant differences were found by sex, sex differences have been identified in visuomotor performance [22, 23, 41]; this factor was accordingly included in the regression analysis. Age has likewise been associated with recovery from concussion [42], and was also included in the analysis. Despite a lack of significance in some of the measures of visuomotor performance, the regression was run on all dependent variables in both conditions, as visuomotor performance has not yet been investigated in both acute concussion and concussion history simultaneously by our group thus far. This was done by comparing performance in the 1st and 3rd sessions (pre-post score = performance in session 1 − performance in session 3) for most dependent variables. For PV only, the pre-post score was calculated by subtracting performance in the 1st session from performance in the 3rd session (performance in session 3 − performance in session 1), as a larger PV in session 3 would indicate improved performance.

### Multivariate regression analyses

In order to tease apart the relative effects of different factors that may contribute to performance recovery, a multivariate hierarchical linear regression resulted in a three-variable model of age, sex, and prior concussion as statistically significant predictors of visuomotor performance. For this analysis, the concussion history and acute concussion groups were combined and regrouped according to the total number of concussions they had previously sustained (1, 2, 3 or more). In the linear regression examining whether age, concussion history (number of concussions), or sex significantly predict visuomotor performance in non-standard tasks, 35 participants were included: 15 healthy controls (7 females, 1 unidentified), 8 adults with a previous history of 1 concussion (7 females), 5 adults with a previous history of 2 concussions (3 females), and 7 adults with a history of 3 or more concussions (3 females). In the analysis examining whether the independent variables predict visuomotor performance on standard tasks, 31 participants were included; 4 participants (1 healthy male, 1 healthy female, and 2 females with a previous history of 3 or more concussions) were excluded due to missing data in this condition.

Results of the final analyses on measures of visuomotor performance (taken as the difference in performance between session 1 and 3) are presented in Additional file 1. A greater difference represents an improvement in visuomotor performance in the 3rd session compared to the first.

#### Movement timing variables

**Fig. 5:**
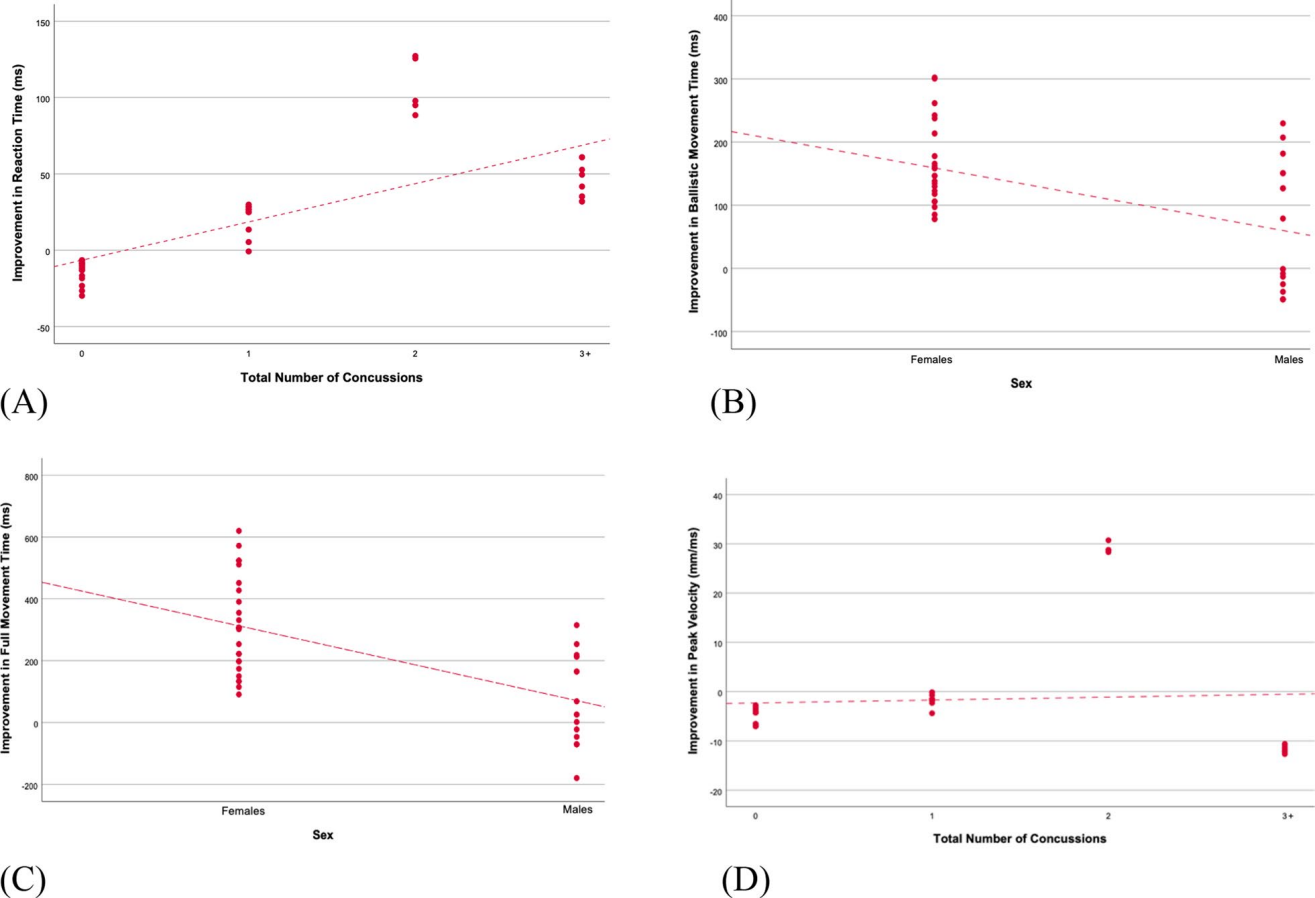
**A** Unstandardized predicted improvement in reaction time over a concussion recovery program in the CMI condition, as a function of number of concussions. Participants with a history of 2 concussions improved their reaction times significantly more than healthy controls. **B** Unstandardized predicted improvements in ballistic and **C** full movement time over a concussion recovery program in the CMI condition, as a function of sex. Females improved their full and ballistic movement times significantly more than males. **D** Unstandardized predicted improvement in peak velocity over a concussion recovery program in the Standard condition, as a function of number of concussions. Participants with a history of 2 concussions improved their peak velocities significantly more than healthy controls. *p < 0.05

RT in the CMI condition was directly associated with concussion history (Fig. 5a; Additional file 1: Fig. 1a). Individuals with 2 concussions experienced an improvement in RT compared to the healthy group, after adjusting for age and sex (ß = 127.64, p = 0.047, R^2^ = 0.117). MTb improvement in the standard condition was directly associated with group. Individuals who had experienced 2 concussions experienced a greater change in visuomotor performance compared to the healthy group, after adjusting for age and sex (ß = 391.51, p = 0.013, R^2^ = 0.269). In the CMI condition, MTb performance was associated with age and sex. Males experienced less improvement in MTb compared to females (ß = − 118.95, p = 0.017, R^2^ = 0.329; Fig. 5b; Additional file 1: Fig. 1b), and there was an increased improvement in visuomotor performance with every year aged (ß = 11.98, p = 0.003, R^2^ = 0.329). There was a significant association between MTf and concussion history in the standard condition: individuals who had sustained 2 concussions experienced a greater improvement in MTf compared to healthy controls, after adjusting for age and sex (ß = 390.28, p = 0.025, R^2^ = 0.251). In the CMI condition, a significant association between age and sex was found after adjusting for concussion history. Male full movement times improved to a lesser extent than female full movement times (ß = − 305.41, p = 0.050, R^2^ = 0.166; Fig. 5c; Additional file 1: Fig. 1c), while there was a significant improvement in visuomotor performance with every year aged (ß = 24.07, p = 0.049, R^2^ = 0.166). A significant association between age, concussion history, and skilled performance was found for PV. In the standard condition, individuals who had sustained 2 concussions experienced a greater improvement in PV compared to healthy controls, after adjusting for sex and age (ß = 33.46, p = 0.013, R^2^ = 0.212; Fig. 5d; Additional file 1: Fig. 1d). In the CMI condition, participants’ improvements in PV increased with every year aged (ß = 1.28, p = 0.035, R^2^ = 0.243). A trend was also observed between PV and number of concussions: individuals who had sustained 2 concussions experienced a greater improvement in PV compared to healthy controls (ß = 22.64, p = 0.062, R^2^ = 0.243).

#### Path length

Concussion history was associated with PLf in the standard condition. Participants who sustained 3 or more concussions improved to a lesser extent than healthy controls (ß = − 4.923, p = 0.047, R^2^ = 0.273). There was an association between PLb and concussion history in the standard condition. After adjusting for age and sex, a trend was observed in which individuals who had sustained 3 or more concussions experienced smaller improvements compared to healthy controls (ß = − 2.88, p = 0.064, R^2^ = 0.199).

#### Absolute error

Absolute error was directly associated with group. Participants who had sustained 3 or more concussions experienced a smaller improvement in the standard condition compared to healthy controls, after adjusting for age and sex (ß = − 1.885, p = 0.029, R^2^ = 0.272). In the CMI condition, individuals who had sustained 2 concussions had a smaller change in visuomotor performance compared to healthy controls (ß = − 4.599, p = 0.017, R^2^ = 0.216), and a trend was observed with sex: male improvements were smaller than female improvements (ß = − 2.159, p = 0.072, R^2^ = 0.216).

#### Variable error

No relationship was found between concussion history, age, or sex and VE performance.

#### Direction reversals

The %DRs in the CMI condition was directly related to age. Individuals experienced a greater change in %DRs every year aged, after adjusting for concussion history and sex (ß = 0.615, p = 0.022, R^2^ = 0.209).

### Pearson correlations

**Fig. 6:**
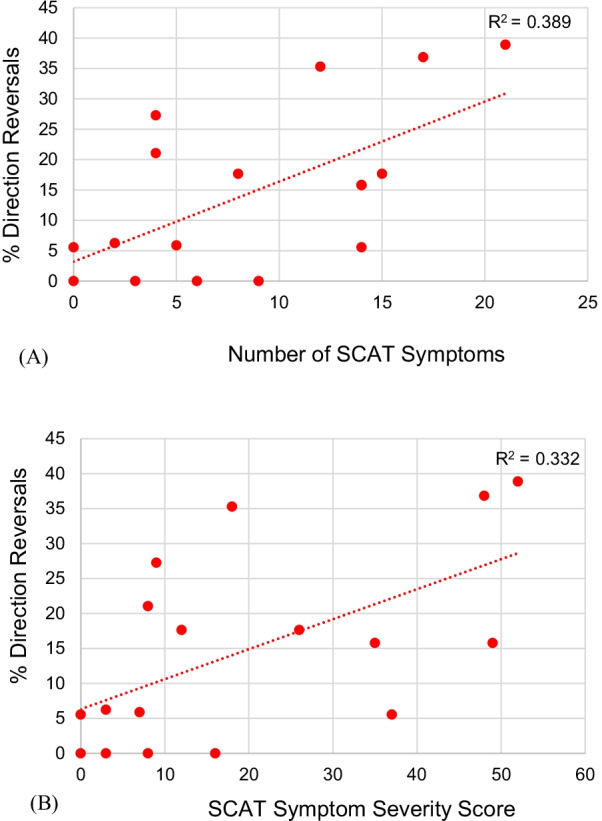
**A** Initial relationship between the % of trials in the CMI condition resulting in a direction reversal and the number of SCAT symptoms. **B** Initial relationship between the % of trials in the CMI condition resulting in a direction reversal and the SCAT symptom severity score. Following the program, there was no longer a relationship between the number of concussion symptoms or severity and CMI performance

Results of the Pearson correlation indicated that there was a significant positive correlation between CMI performance deficits (%DRs) and number of concussion symptoms prior to beginning the recovery program (r = 0.624, N = 17, p = 0.007; Fig. 6a). Following the recovery program, this relationship was no longer significant (r = 0.388, N = 13, p = 0.190). Similarly, a significant positive correlation was identified between CMI performance deficits (%DRs) and severity of concussion symptoms prior to beginning the recovery program (r = 0.576, N = 17, p = 0.015; Fig. 6b). Again, the relationship was no longer significant following completion of the program (r = 0.322, N = 13, p = 0.284).

## Discussion

The present study sought to determine whether an active recovery program was associated with improved visuomotor performance in acutely concussed adults and adults with a history of concussion compared to healthy no-history controls. A secondary aim of this study was to assess whether CMI deficits may be related to sex and age. Our findings indicate that performance on tasks requiring visuomotor integration may improve over recovery. We observed differences in performance on complex visuomotor tasks between acutely concussed individuals, individuals with a history of concussion, and healthy controls with no history of concussion. Specifically, participants who were acutely concussed had difficulty executing visually guided movements when there was a dissociation between the guiding visual information and the required motor action prior to the recovery program. These difficulties included deficits in movement planning and execution. However, following the program, participants with a history of concussion experienced larger improvements in performance on several visuomotor measures, as indicated by larger differences in performance between the first and final session, though there remained lingering deficits in some measures. Moreover, we observed sex-related differences in the performance of complex visuomotor tasks, with females outperforming males on the basis of movement timing variables. Finally, a relationship between age and improved performance on complex, rule-based tasks was found.

The results of the current study demonstrate that in both healthy individuals and individuals affected by concussion, performance of a rule-based movement task can be improved over time. Our findings also reveal that prior to a concussion recovery program, movement timing and accuracy in tasks requiring CMI are poor in those acutely affected by concussion compared to non-concussed adults with a history of concussion and healthy adults with no prior concussion history. However, following the program, movements were faster and more accurate. These results complement findings from our previous studies with working aged healthy adults and adults at-risk for the development of Alzheimer’s disease (AD), in which both groups experienced improvements in visuomotor performance [43, 44]. In combination with the findings from the current study, this suggests that both in the presence and absence of clinically altered brain function, visuomotor training may be beneficial for generalized improvement of functional ability. The ability to perform complex, rule-based tasks is crucial for many daily life and sport-related activities, and is impaired in several neurological conditions [17, 19, 20, 41]. Our results support the notion that this important ability can be improved. It is likely that improvements in visuomotor performance occurred as a result of both time and treatment; thus, future work with a delayed-treatment control group will be investigating this possibility. The potential for improving motor functions requiring cognitive-motor integration is particularly important in individuals affected by brain dysfunction.

Our findings suggest the existence of visuomotor deficits in acutely concussed adults. As in our previous studies, the visuomotor deficits were evident when there was a dissociation between vision and action, and were observed in movement execution and movement accuracy [17, 19]. Prior to the recovery program, the CMI task appears to bring out behavioural deficits in acutely concussed participants. We suggest that these deficits may be due to disruptions in frontoparietal networks and in communication between brain regions responsible for the planning and execution of cognitive-motor integration. These deficits may likewise be due to altered integrity of frontoparietal-cerebellar white matter tracts—the cerebellum is heavily involved in CMI, given the need for online predictive movement feedback in the performance of these tasks [10, 45]. In fact, studies have reported frontoparietal-cerebellar network changes post-concussion. In particular, for networks that may be specifically related to CMI, studies with concussed individuals have found increased activation in parietal, frontal, and cerebellar regions when compared with pre-injury functional magnetic resonance imaging (fMRI) data [9, 15, 46–50]. Moreover, anatomical studies have found altered diffusion characteristics within white matter tracts in concussed adolescents, in addition to younger and older adults with concussion history, in the pathways connecting frontal and parietal regions [49, 51–53], as well as an association between white matter integrity along frontoparietal-cerebellar white matter tracts and visuomotor performance in individuals affected by persistent concussion symptoms [42, 44]. These data are in line with the current findings demonstrating impairment in tasks requiring CMI following concussion, suggesting that a crucial role is played by frontoparietal-cerebellar networks on tasks integrating thought and action [46, 48, 54, 55]. In the current study, the acutely concussed group improved over the course of the program while performance of other groups remained steady. This suggests that improvements in visuomotor performance may be related to the recovery of rule-based movement control brain networks. Future studies incorporating the use of fMRI, electroencephalography (EEG), and functional near-infrared spectroscopy (fNIRS) would provide additional insight into the neural correlates of impaired performance, and into the underlying recovery of brain networks involved in the performance of complex, rule-based tasks following concussion.

Despite initial deficits compared to the healthy group, participants with a history of concussion experienced greater improvements in both standard tasks and tasks requiring CMI compared to healthy controls following the recovery program. Notably, improvements in movement timing and movement execution variables primarily occurred in individuals who had sustained 2 concussions, while participants who had sustained 1 or 3 or more concussions demonstrated either no improvements or decreased improvements compared to healthy controls. Our findings suggest an important role of number of concussions sustained in the ability to recover performance of complex visuomotor tasks. Multiple concussions have been associated with cognitive and motor deficits, psychiatric impairments, neurodegenerative diseases, and impaired recovery, though most of the literature investigates these effects in athletes [56, 57]. Studies have reported that across the lifespan, prior concussion history is associated with less recovery in athletes compared to athletes sustaining their first concussion [58]. Moreover, history of multiple concussions, generally defined as 2 or more concussions, is associated with altered balance and gait characteristics, in addition to prolonged neurocognitive and symptom recovery, as indicated by larger reaction times and lower memory performance [57, 59–62]. Impairments associated with multiple concussions in athletes may be due to electrophysiological changes and disrupted communication between brain areas involved in the performance of these tasks. In fact, studies have demonstrated supressed electrophysiological activity in asymptomatic multiple-concussion athletes compared to healthy controls, after adjusting for time since the latest concussion [63, 64]. Similarly, functional connectivity in the anterior default mode network is significantly lower after sustaining multiple concussions compared to sustaining 1 concussion [65]. Though our data demonstrate that improved CMI performance occurs in those with a history of 2 concussions, prior studies do not differentiate between 2 and 3 or more concussions. Our findings support the notion that multiple concussions may impair recovery, as evidenced by diminished visuomotor performance improvements in individuals who sustained 3 or more concussions compared to healthy controls, though we further distinguish that sustaining 1 concussion may result in comparable performance to healthy controls, while 2 concussions may allow the ability to recover performance deficits. Thus, there may be a ‘dose–response’ relationship between the number of concussions and the ability to recover cognitive-motor function. More research is needed in the non-athlete population in order to further support this notion.

Sex-related differences and an effect of age were observed in this study. After concussion history and age were adjusted for, male movement times in the standard and CMI conditions improved to a lesser extent than female movement times. This is consistent with a previous study from our group demonstrating that males performed worse in a CMI task [41]. Furthermore, previous studies have observed that both CMI and standard tasks evoke a notably more bilateral pattern of activity in premotor and parietal regions in women compared to men, though in CMI tasks, men have greater lateral sulcus activity than women [8, 22]. Thus, differences in brain activation patterns may contribute to observed differences in the current study, though it is also likely that since males typically perform worse in visuomotor tasks, there is the potential for stronger recovery on CMI measures in females compared to males.

Finally, in the CMI condition, aging was associated with a greater improvement in performance. Though visuomotor performance declines are typically associated with aging [41, 43, 44], our study investigated performance in young to working-aged adults. Therefore, performance declines would not be expected to occur with age in this group. In younger age groups, adolescents demonstrate improved performance in CMI tasks compared to children (11–12 years old) and young children (8–10 years old) [19]. Moreover, children and adolescents with concussion history perform worse than young adults with concussion history [19]. Taken together, the evidence suggests that visuomotor performance follows an inverted U-shaped curve in aging, during which growing and aging brains are neurologically more fragile for executing CMI tasks. This may explain the mechanism underlying the improvement associated with age in the current study: younger participants’ brain networks were more fragile than older ones. Thus, working-aged adults in this study may have stronger networks controlling the performance of complex, rule-based movements, and this may underlie the greater improvements seen with age.

Sensorimotor integration is essential to the performance of complex, rule-based tasks. Performance on such tasks is impaired by concussion, and it is therefore essential to incorporate recovery of visuomotor skill performance in concussion management. The goal of the present study was to examine the recovery of CMI in concussed working-aged adults and adults with a history of concussion compared with healthy no-history controls during the course of a post-concussion management program. Prior to the program, individuals acutely affected by concussion demonstrated impaired performance on complex visuomotor tasks. When the acute concussion and concussion history groups were combined, it was observed that greater improvements occurred in participants with a history of 2 concussions compared to no-concussion-history healthy controls over the course of the program. Moreover, females demonstrated greater improvement of these skills compared to males, and a positive effect of age was observed. These data suggest that the number of concussions sustained affects the integrity of brain networks controlling skilled performance, and that the underlying brain networks that control cognitive-motor integration are different between males and females. These differences should be further investigated in a broader age range, as these results provide important factors to consider in concussion management.

### Strengths and limitations

The current study investigated changes in visuomotor performance in individuals who are acutely concussed, those with a history of concussion, and healthy controls. Individuals who are acutely concussed or have a history of concussion are rarely studied simultaneously. As concussion is a heterogeneous condition, a key strength of this study was to investigate performance differences between these 2 groups separately and healthy controls. In addition, studies investigating concussion typically examine elite athletes. The inclusion of participants from the community who engage in all exercise levels in the current study indicates that the findings may be more generalizable than studies only considering elite athletes.

When interpreting the results of this study, it is important to acknowledge that the assessment of past concussion history was based on self-report. Therefore, potential errors are possible due to imprecise memory. Moreover, not all individuals seek medical care in the face of concussion, and consequently may not receive a concussion diagnosis. Thus, individuals who were placed in the healthy group may have belonged in the concussion or concussion history groups. In addition, a larger sample would be helpful for investigating the relationship between sex-related differences and concussion. Furthermore, the lack of a control group or a group receiving an alternate treatment excludes the ability to examine whether observed differences in this study were associated with OMT. Finally, as the CMI task was repeated during each session, there is a possibility that some of the observed improvements were due to learning effects. However, only the concussion group demonstrated decreases in CMI variables during the course of the recovery program, and when the acute concussion and concussion history groups were combined, a history of 2 concussions predicted greater improvements in visuomotor performance compared to healthy-no-history controls, suggesting that improvements primarily due to learning effects are minimal.

## Conclusions

The results of the present study demonstrate an overall improvement of visuomotor performance over the course of an active recovery program involving OMT, indicating that skilled performance on tasks requiring CMI may be improved. Moreover, prior to the program, individuals with an acute concussion showed distinct impairments in the performance of CMI tasks. Following the program, performance on these tasks improved. When previous number of concussions was investigated in both acutely concussed participants and non-concussed participants with a history of concussion, it was revealed that greater improvements in visuomotor tasks occurred in individuals with a concussion history compared to healthy controls. Taken together, this suggests that eye-hand coordination is impaired following mild brain injury, though recovery is possible and may be related to age. Additionally, the results suggest that underlying brain networks controlling simultaneous thought and action may differ between sexes. Overall, these findings contribute to present knowledge about motor performance on tasks requiring rule integration.

## Supplementary Information

**Additional file 1: Supplementary Table 1.** Association between visuomotor performance and history of concussion, sex, and age. **Supplementary Figure 1.** (**A**) Mean improvement in reaction time over a concussion recovery program in the CMI condition, as a function of number of concussions. Participants with a history of 2 concussions improved their reaction times significantly more than healthy controls. (**B**) Mean improvements in ballistic and (**C**) full movement time over a concussion recovery program in the CMI condition, as a function of sex. Females improved their full and ballistic movement times significantly more than males. (**D**) Mean improvement in peak velocity over a concussion recovery program in the Standard condition, as a function of number of concussions. Participants with a history of 2 concussions improved their peak velocities significantly more than healthy controls. *: *p *< 0.05.

## Data Availability

The raw datasets generated and/or analysed during the current study are not publicly available due to confidential health information about the participants but are available from the corresponding author in an anonymized form on reasonable request.

## Abbreviations

AE: Absolute error
AD: Alzheimer’s disease
CMI: Cognitive-motor integration
EEG: Electroencephalography
fMRI: Functional magnetic resonance imaging
fNIRS: Functional near-infrared spectroscopy
MRI: Magnetic resonance imaging
MT: Movement time
MTf: Full movement time
MTb: Ballistic movement time
mTBI: Mild traumatic brain injury
OMT: Osteopathic manual treatment
PL: Path length
PLf: Full path length
PLb: Ballistic path length
PV: Peak velocity
RT: Reaction time
SD: Standard deviation
VE: Variable error
%DR: Percentage of direction reversals
