# Supplementary material for: Sex-related differences in visuomotor skill recovery following concussion in working-aged adults
Source: BMC Sports Sci Med Rehabil. 2022 Apr 20;14:72. doi: 10.1186/s13102-022-00466-6 (PMC9022305; doi:10.1186/s13102-022-00466-6)
Supplement: Supplementary file 1 — Additional file 1: Supplementary Table 1. Association between visuomotor performance and history of concussion, sex, and age. Supplementary Figure 1. (A) Mean improvement in reaction time over a concussion recovery program in the CMI condition, as a function of number of concussions. Participants with a history of 2 concussions improved their reaction times significantly more than healthy controls. (B) Mean improvements in ballistic and (C) full movement time over a concussion recovery program in the CMI condition, as a function of sex. Females improved their full and ballistic movement times significantly more than males. (D) Mean improvement in peak velocity over a concussion recovery program in the Standard condition, as a function of number of concussions. Participants with a history of 2 concussions improved their peak velocities significantly more than healthy controls. *: p < 0.05. [file 13102_2022_466_MOESM1_ESM.docx]

**Supplementary Table 1:** Association between visuomotor performance and history of concussion, sex, and age.

| VARIABLES AFFECTING NON-STANDARD VISUOMOTOR PERFORMANCE (CMI Condition)  N = 35 | | | | |
| --- | --- | --- | --- | --- |
|  | **Model 1 (R^2^ = 0.209)** | | | |
| % Direction Reversal |  | **Unstandardized B** | **95% CI** | **p value** |
|  |  | **Unstandardized B** | **95% CI** | **p value** |
|  | **Age** | 0.615 | 0.092, 1.138 | **0.022** |
|  | **1 Concussionº** | -2.995 | -11.127, 5.138 | 0.46 |
|  | **2 Concussionsº** | 2.444 | -8.078, 12.966 | 0.64 |
|  | **3+ Concussionsº** | 3.414 | -4.918, 11.745 | 0.411 |
|  | **Male†** | -4.197 | -10.853, 2.459 | 0.209 |
| Reaction Time (ms) | **Model 1 (R^2^ = 0.117)** | | | |
|  |  | **Unstandardized B** | **95% CI** | **p value** |
|  | **Age** | -1.633 | -7.872, 4.607 | 0.599 |
|  | **1 Concussionº** | 33.561 | -63.489, 130.611 | 0.488 |
|  | **2 Concussionsº** | 127.642 | 2.076, 253.207 | **0.047** |
|  | **3+ Concussionsº** | 61.34 | -38.085, 160.764 | 0.219 |
|  | **Male†** | -2.851 | -82.282, 76.580 | 0.942 |
| Full Movement Time (ms) | **Model 1 (R^2^ = 0.166)** | | | |
|  |  | **Unstandardized B** | **95% CI** | **p value** |
|  | **Age** | 24.071 | 0.060, 48.083 | **0.049** |
|  | **1 Concussionº** | -85.1 | -458.854, 288.383 | 0.647 |
|  | **2 Concussionsº** | -95.846 | -579.069, 387.378 | 0.69 |
|  | **3+ Concussionsº** | -53.434 | -436.056, 329.188 | 0.779 |
|  | **Male†** | -305.414 | -611.092, 0.265 | **0.05** |
| Ballistic Movement Time (ms) | **Model 1 (R^2^ = 0.329)** | | | |
|  |  | **Unstandardized B** | **95% CI** | **p value** |
|  | **Age** | 11.975 | 4.438, 19.512 | **0.003** |
|  | **1 Concussionº** | 52.637 | -64.595, 169.869 | 0.369 |
|  | **2 Concussionsº** | 39.144 | -112.534, 190.821 | 0.604 |
|  | **3+ Concussionsº** | 32.158 | -87.943, 152.258 | 0.59 |
|  | **Male†** | -118.954 | -214.903, -23.005 | **0.017** |
| Full Path Length (mm) | **Model 1 (R^2^ = 0.192)** | | | |
|  |  | **Unstandardized B** | **95% CI** | **p value** |
|  | **Age** | 1.417 | -0.265, 3.099 | 0.095 |
|  | **1 Concussionº** | -19.651 | -47.977, 8.674 | 0.166 |
|  | **2 Concussionsº** | -23.167 | -55.731, 9.397 | 0.156 |
|  | **3+ Concussionsº** | -9.568 | -37.644, 18.509 | 0.491 |
|  | **Male†** | -21.379 | -44.420, 1.662 | 0.068 |
| Ballistic Path Length (mm) | **Model 1 (R^2^ = 0.141)** | | | |
|  |  | **Unstandardized B** | **95% CI** | **p value** |
|  | **Age** | 1.177 | -0.239, 2.593 | 0.1 |
|  | **1 Concussionº** | -11.673 | -33.694, 10.347 | 0.289 |
|  | **2 Concussionsº** | -18.234 | -46.724, 10.257 | 0.203 |
|  | **3+ Concussionsº** | -8.332 | -30.891, 14.227 | 0.459 |
|  | **Male†** | -14.531 | -32.553, 3.492 | 0.111 |
| Absolute Error (mm) | **Model 1 (R^2^ = 0.216)** | | | |
|  |  | **Unstandardized B** | **95% CI** | **p value** |
|  | **Age** | 0.15 | -0.035, 0.336 | 0.109 |
|  | **1 Concussionº** | -0.87 | -3.754, 2.014 | 0.544 |
|  | **2 Concussionsº** | -4.599 | -8.330, -0.867 | **0.017** |
|  | **3+ Concussionsº** | -0.736 | -3.691, 2.218 | 0.616 |
|  | **Male†** | -2.159 | -4.519, 0.202 | 0.072 |
| Variable Error (mm) | **Model 1 (R^2^ = 0.091)** | | | |
|  |  | **Unstandardized B** | **95% CI** | **p value** |
|  | **Age** | 0.024 | -0.015, 0.062 | 0.221 |
|  | **1 Concussionº** | 0.067 | -0.531, 0.665 | 0.823 |
|  | **2 Concussionsº** | 0.297 | -0.477, 1.07 | 0.442 |
|  | **3+ Concussionsº** | -0.164 | -0.776, 0.449 | 0.592 |
|  | **Male†** | -0.132 | -0.621, 0.358 | 0.588 |
| Peak Velocity (mm/ms) | **Model 1 (R^2^ = 0.243)** | | | |
|  |  | **Unstandardized B** | **95% CI** | **p value** |
|  | **Age** | 1.276 | 0.092, 2.46 | **0.035** |
|  | **1 Concussionº** | 9.998 | -8.415, 28.41 | 0.278 |
|  | **2 Concussionsº** | 22.641 | -1.182, 46.464 | 0.062 |
|  | **3+ Concussionsº** | 5.528 | -13.335, 24.391 | 0.556 |
|  | **Male†** | 1.026 | -14.044, 16.096 | 0.891 |
| VARIABLES AFFECTING STANDARD VISUOMOTOR PERFORMANCE (Standard Condition)  N = 31 | | | | |
| Reaction Time (ms) | **Model 1 (R^2^ = 0.063)** | | | |
|  |  | **Unstandardized B** | **95% CI** | **p value** |
|  | **Age** | 1.223 | -4.717, 7.163 | 0.679 |
|  | **1 Concussionº** | -10.907 | -103.295, 81.482 | 0.812 |
|  | **2 Concussionsº** | -57.344 | -176.880, 62.191 | 0.337 |
|  | **3+ Concussionsº** | 19.979 | -74.670, 114.629 | 0.671 |
|  | **Male†** | 25.059 | -50.557, 100.675 | 0.506 |
| Full Movement Time (ms) | **Model 1 (R^2^ = 0.251)** | | | |
|  |  | **Unstandardized B** | **95% CI** | **p value** |
|  | **Age** | 5.21 | -11.612, 22.031 | 0.534 |
|  | **1 Concussionº** | 10.571 | -251.077, 272.220 | 0.935 |
|  | **2 Concussionsº** | 390.277 | 51.749, 728.805 | **0.025** |
|  | **3+ Concussionsº** | -138.225 | -406.276, 129.826 | 0.303 |
|  | **Male†** | -134.857 | -349.004, 79.290 | 0.21 |
| Ballistic Movement Time (ms) | **Model 1 (R^2^ = 0.269)** | | | |
|  |  | **Unstandardized B** | **95% CI** | **p value** |
|  | **Age** | 4.884 | -10.281, 20.049 | 0.518 |
|  | **1 Concussionº** | -21.026 | -256.908, 214.856 | 0.858 |
|  | **2 Concussionsº** | 391.507 | 86.316, 696.698 | **0.013** |
|  | **3+ Concussionsº** | -101.984 | -343.638, 139.670 | 0.398 |
|  | **Male†** | -122.994 | -316.052, 70.064 | 0.205 |
| Full Path Length (mm) | **Model 1 (R^2^ = 0.273)** | | | |
|  |  | **Unstandardized B** | **95% CI** | **p value** |
|  | **Age** | -0.149 | -0.422, 0.125 | 0.273 |
|  | **1 Concussionº** | -0.767 | -5.065, 3.530 | 0.716 |
|  | **2 Concussionsº** | -0.223 | -5.118, 4.671 | 0.926 |
|  | **3+ Concussionsº** | -4.923 | -9.776, -0.071 | **0.047** |
|  | **Male†** | -1.002 | -4.763, 2.759 | 0.587 |
| Ballistic Path Length (mm) | **Model 1 (R^2^ = 0.199)** | | | |
|  |  | **Unstandardized B** | **95% CI** | **p value** |
|  | **Age** | -0.133 | -0.324, 0.059 | 0.17 |
|  | **1 Concussionº** | -0.677 | -3.661, 2.306 | 0.648 |
|  | **2 Concussionsº** | -0.023 | -3.883, 3.838 | 0.991 |
|  | **3+ Concussionsº** | -2.88 | -5.937, 0.176 | 0.064 |
|  | **Male†** | -1.295 | -3.737, 1.147 | 0.289 |
| Absolute Error (mm) | **Model 1 (R^2^ = 0.272)** | | | |
|  |  | **Unstandardized B** | **95% CI** | **p value** |
|  | **Age** | -0.076 | -0.181, 0.029 | 0.152 |
|  | **1 Concussionº** | -1.057 | -2.693, 0.579 | 0.198 |
|  | **2 Concussionsº** | 1.024 | -1.092, 3.140 | 0.333 |
|  | **3+ Concussionsº** | -1.885 | -3.560, -0.209 | **0.029** |
|  | **Male†** | -0.795 | -2.133, 0.544 | 0.237 |
| Variable Error (mm) | **Model 1 (R^2^ = 0.111)** | | | |
|  |  | **Unstandardized B** | **95% CI** | **p value** |
|  | **Age** | 0.014 | -0.018, 0.047 | 0.376 |
|  | **1 Concussionº** | 0.266 | -0.240, 0.771 | 0.294 |
|  | **2 Concussionsº** | 0.163 | -0.491, 0.818 | 0.616 |
|  | **3+ Concussionsº** | 0.396 | -0.122, 0.914 | 0.13 |
|  | **Male†** | -0.17 | -0.584, 0.244 | 0.41 |
| Peak Velocity (mm/ms) | **Model 1 (R^2^ = 0.212)** | | | |
|  |  | **Unstandardized B** | **95% CI** | **p value** |
|  | **Age** | 0.116 | -1.178, 1.411 | 0.856 |
|  | **1 Concussionº** | 2.741 | -17.4, 22.881 | 0.784 |
|  | **2 Concussionsº** | 33.46 | 7.402, 59.519 | **0.013** |
|  | **3+ Concussionsº** | -6.584 | -27.217, 14.05 | 0.522 |
|  | **Male†** | -2.409 | -18.894, 14.075 | 0.769 |

Blocked hierarchical linear regression model 1 adjusts for age, history of concussion, and sex

° Reference category = healthy adults (no history of concussion).

^†^ Males compared to females.

For each unit of the participants’ characteristics there is on average an increase or a decrease in unstandardized B, representing the change in visuomotor performance scores (pre-recovery program score – post-recovery program score). A positive unstandardized B indicates a greater change in participants’ scores (an improvement) and a negative unstandardized B indicates a smaller change in participants’ scores.

**Supplementary Figure 1:**


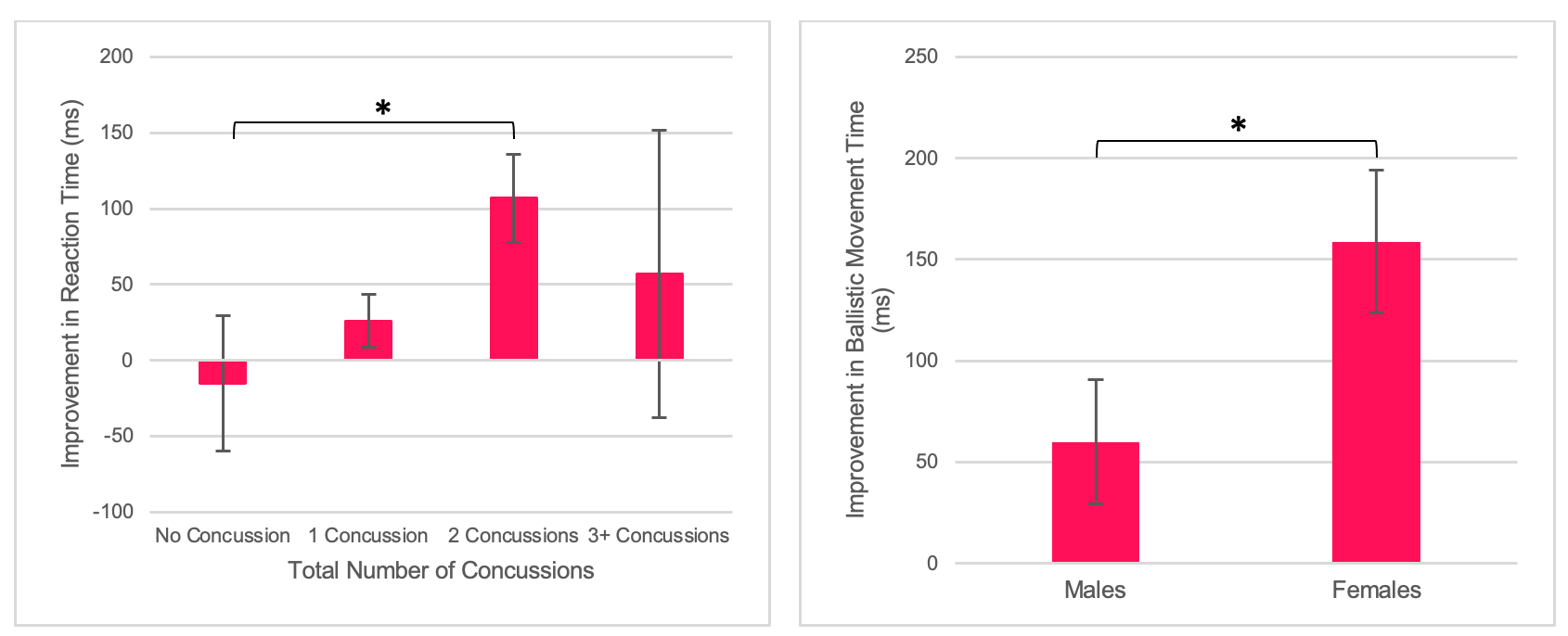


1. (B)


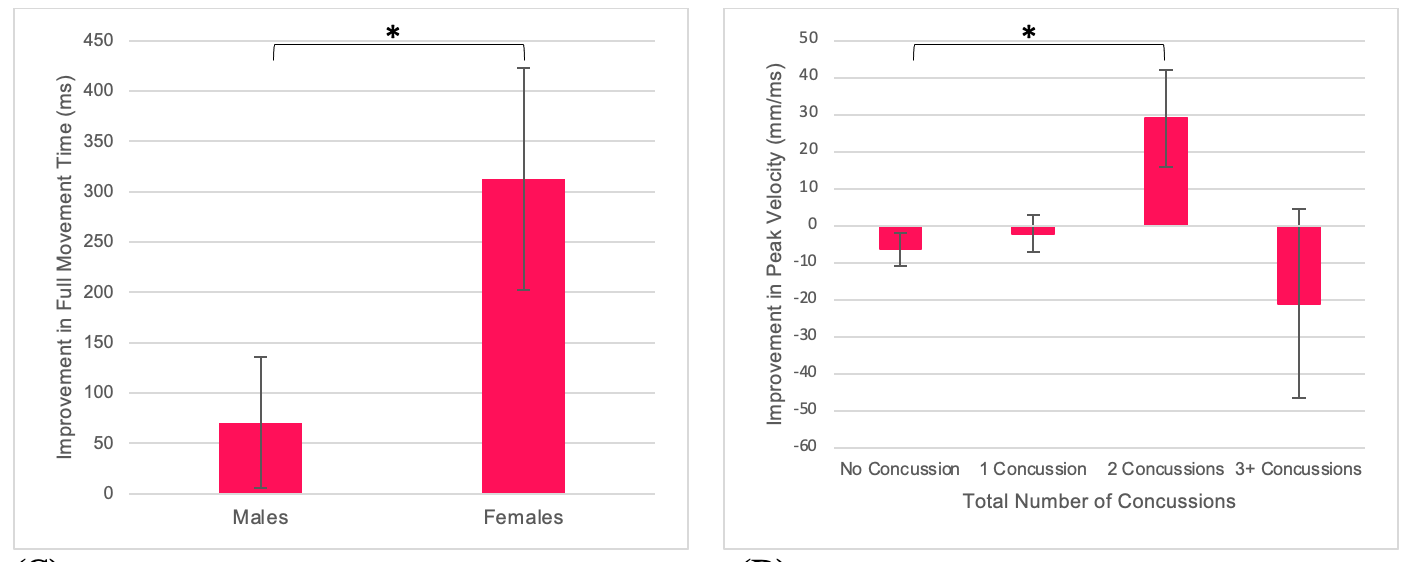


(C) (D)

Supplementary Figure 1. (A) Mean improvement in reaction time over a concussion recovery program in the CMI condition, as a function of number of concussions. Participants with a history of 2 concussions improved their reaction times significantly more than healthy controls. (B) Mean improvements in ballistic and (C) full movement time over a concussion recovery program in the CMI condition, as a function of sex. Females improved their full and ballistic movement times significantly more than males. (D) Mean improvement in peak velocity over a concussion recovery program in the Standard condition, as a function of number of concussions. Participants with a history of 2 concussions improved their peak velocities significantly more than healthy controls. *: p<0.05
